# Social inequality and the risk of living in a nursing home: implications for the COVID-19 pandemic

**DOI:** 10.1186/s41118-021-00119-5

**Published:** 2021-06-23

**Authors:** Fabrizio Bernardi, Marco Cozzani, Francesca Zanasi

**Affiliations:** 1 European University Institute, San Domenico di Fiesole, Italy; 2 University of Bari Aldo Moro, Bari, Italy

## Abstract

Across EU countries, all available evidence suggests that the number of deaths linked to COVID-19 among those living in nursing homes has been extremely high. However, it is largely unknown to what extent income and education affect the probability of being a nursing home resident. If the probability of residing in a nursing home is stratified by socio-economic status, under the current COVID-19 pandemic socio-economic inequality in the probability of living in a nursing home could contribute to enlarge socio-economic inequalities in the risk of mortality with COVID-19. In this article, we investigate whether there are income and educational differences in the likelihood of being a resident in a nursing home across 12 European countries. We use SHARE data (waves 5–7) and compute logistic regression models for rare events. We find that low-educated individuals and those having household income below the national median are more likely to live in a nursing home. This general pattern holds across all the European countries considered. However, there is considerable uncertainty in our estimates due to a small sample size, and firm conclusions on how the effect of socio-economic characteristics varies across countries cannot be drawn. Still, there is some indication that educational and income differences are the largest in the Scandinavian countries (Denmark and Sweden) and the Netherlands, while the smallest ones are found in Italy, with the remaining countries laying in between.

## Introduction

All available evidence suggests that the number of deaths for COVID-19 among those living in nursing and care homes^1^ has been extremely high, being between 30% and 60% of overall COVID-related deaths in Europe during the spring of 2020 (Danis et al., 2020)^2^. For example, between March and May 2020, during the first wave of COVID-19 infections, in Belgium and France about half of deaths linked to COVID-19 happened in nursing homes (Danis et al., 2020; Frijters & van Uffelen, 2020; Orange, 2020). Moreover, despite the introduction of rigid protocols to protect nursing home residents, the number of deaths with COVID-19 in nursing homes has been extremely high also during the second wave in the fall of 2020 in all European countries (ECDC, 2020).

Nursing homes usually host elderly individuals with pre-existing health conditions and an extra need of support with personal care. Moreover, nursing home residents share common spaces and have daily contacts with the same caring assistants. Altogether, these circumstances increase their risk of being exposed to COVID-19 and to develop its most severe symptoms. In case of illness, the quality of medical care in nursing homes is generally inferior compared to the one provided in a hospital. All the abovementioned factors (pre-existing health conditions, shared spaces, and relatively lower quality medical care) made nursing homes a critical trouble spot during the COVID-19 pandemic.

Against the backdrop of this major public health disaster that occurred in nursing homes, in this article, we investigate whether there are educational and income differences in the likelihood of living in a nursing home in Europe at time of COVID-19 pandemic. In doing this, we also contribute to the literature on socio-economic inequalities in the risk of death with COVID-19 (Strang, Fürst, & Schultz, 2020). From previous studies, we know that there is a socio-economic gradient in the risk of death by COVID-19 with a higher risk for low socio-economic status groups, and, as noted above, that those who live in a nursing home are particularly exposed to the risk of death with COVID-19. What we do not know is to what extent the extremely high number of deaths among nursing home residents during the COVID-19 pandemic has widened the socio-economic gradient in the risk of death with COVID-19. By showing the socio-economic composition of the residents in nursing homes (who, as we know, have also been highly exposed to the risk of death during the COVID-19 pandemic), we make the first step to fill this gap in the literature.

In our empirical analyses, we use data from the 2013–2017 waves of the Survey of Health, Ageing, and Retirement in Europe (SHARE) and we focus on individuals aged 65 and older living in 12 European countries: Austria, Belgium, Czech Republic, Denmark, France, Germany, Italy, The Netherlands, Slovenia, Spain, Sweden, and Switzerland.

A previous study based on SHARE data between 2007 and 2011 already suggested that in Europe, moving into a nursing home is more frequent among those with low wealth and income (Laferrère, Van Den Heede, & Van Den Bosch, 2012). With respect to that study, our contribution is threefold. We use more recent data from 2013 to 2017, consider a larger number of countries, and present results separately for each country. To the best of our knowledge, no study has addressed country-specific socio-economic inequalities in access to a nursing home so far. In this way, we provide a country-specific snapshot of socio-economic differences in the probability of living in nursing homes in very recent years and, thus, indirectly of socio-economic inequalities in the risk of being exposed to the COVID-19 epidemic within care homes in 2020.

In the next section, we first present our analytical framework. Specifically, we posit our study about the socio-economic differences of living in a nursing home as a part of a larger framework, informative on the (age-specific) risk of dying by COVID-19 in a nursing home and related socio-economic inequalities. Afterwards, we briefly discuss cross-country differences in long-term care (LTC) systems and possible determinants of inequalities in living in a nursing home. In the third section, we describe data, variables, and method used for the analysis. In the fourth section, we present our main findings on socio-economic inequalities in the probability of living in a nursing home based on SHARE data. In the conclusion, we discuss the more general implication of our findings for studies on social inequality and the COVID-19 pandemic.

## An analytical framework

The overall interest of this article is on social inequality in the age-specific risk of dying with COVID-19 for those living in a nursing home (P_age)_. Ideally, we would compare P_age_ (dying with COVID in a nursing home - NH) for those of high and low SES. Since the information on the SES of those who died with COVID is not available in any European data source, we can use an indirect approach and factor P_age_ (dying with COVID in a NH) as the following chain:

1 $$ {\displaystyle \begin{array}{c}{\mathrm{P}}_{\mathrm{age}}\left(\mathrm{dying}\ \mathrm{with}\ \mathrm{COVID}\ \mathrm{in}\ \mathrm{a}\ \mathrm{NH}\right)=\\ {}{\mathrm{P}}_{\mathrm{age}}\left(\mathrm{living}\ \mathrm{in}\ \mathrm{a}\ \mathrm{nursing}\ \mathrm{home}\right)\times \end{array}} $$

2 $$ {\mathrm{P}}_{\mathrm{age}}\left(\mathrm{being}\ \mathrm{in}\mathrm{fected}\ \mathrm{with}\ \mathrm{COVID}\ \mathrm{in}\ \mathrm{a}\ \mathrm{NH}|\mathrm{living}\ \mathrm{in}\ \mathrm{a}\ \mathrm{NH}\right)\times $$

3 $$ {\mathrm{P}}_{\mathrm{age}}\left(\mathrm{dying}\ \mathrm{with}\ \mathrm{COVID}\ \mathrm{in}\ \mathrm{a}\ \mathrm{NH}|\mathrm{being}\ \mathrm{in}\mathrm{fected}\ \mathrm{with}\ \mathrm{COVID}\ \mathrm{in}\ \mathrm{a}\ \mathrm{NH}\right) $$

In this article, we will focus on the first probability in the chain (1) and study socio-economic inequality in the probability of living in a NH. We can however comment shortly also on the two other probabilities. Firstly, the segregated nature of the population in the NH sharing common space and being cared after by the same staff has made the probability of infection very high. Secondly, the fragile health conditions of those living in NH have also made for a very high risk of death among those infected. The second and third terms in the chain of probabilities are therefore critical to explain the high risk of death in the NH during the COVID-19 pandemic. An important question for the purpose of this article is, then, whether there might be socioeconomic inequalities in the second and third components of chain of probabilities. In this respect, if high and low SES individuals lived in the same NH, it seems reasonable to assume that they run the same risk of infection and death. Conversely, if high and low SES subjects lived in different NHs that were segregated by SES, one might hypothesize that high SES NHs were better equipped to reduce both the risk of infection and risk of death in case of being infected. We are not aware of neither academic nor anecdotal evidence that suggests accordingly. In any case, even if that segregated scenario favorable to high SES residents in NH were true, social inequalities eventually found in a component (1) would be accentuated by those found in components (2) and (3).

## Background

### Cross-country difference in long-term care systems

European countries differ in the characteristics of their LTC systems. Overall, European LTC systems can be classified over a continuum from the *informal care-led model*, to the *services-led model*, with Mediterranean countries placed on the first extreme, Scandinavian countries on the opposite pole (Pavolini & Ranci, 2008) and Central European countries somewhere in the middle (Gori, Fernandez, & R., 2015; Haberkern, Schmid, Neuberger, & Grignon, 2011)*.* Eastern European countries tend to resemble the first side of the spectrum (Colombo, Llena-Nozal, Mercier, & Tjadens, 2011). The informal care-led model largely relies on family members that provide care to their elderly relatives. The state only compensates part of the costs of dependency with cash transfers and care allowances (Jessoula et al. 2018). The public funding of long-term institutional care, i.e., care provided within licensed facilities, is very limited, resulting in a scarcity of beds available in nursing and care homes. In the service-led model, higher public spending in long-term institutional care is connected with a large number of nursing homes in place to relieve the family from such responsibilities.

**Fig. 1:**
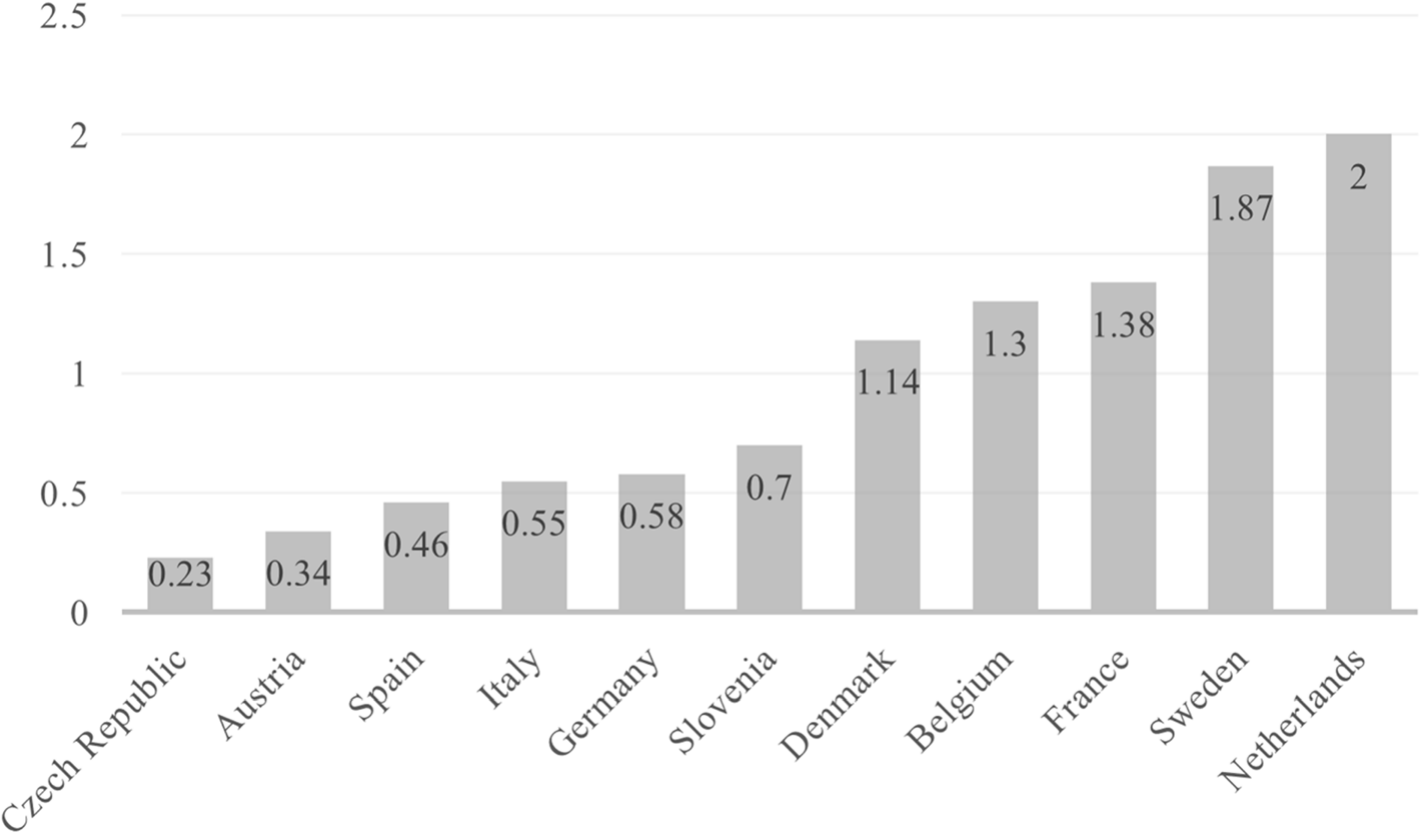
Public expenditure on LTC in institutions as % of GDP, 2010. Data for Switzerland not available. Source: Lipszyc, Sail, & Xavier (2012)

Different logics of LTC lead to large variation both in public spending in LTC institutions and in the supply of services. As far as public spending is concerned (Fig. 1), the Netherlands, Sweden, France, and Belgium devote between 1.5 and 2 percent of the national GDP to institutional care, while the share is much lower in Italy and Spain, but also in Germany, Austria, and the Czech Republic.

**Fig. 2:**
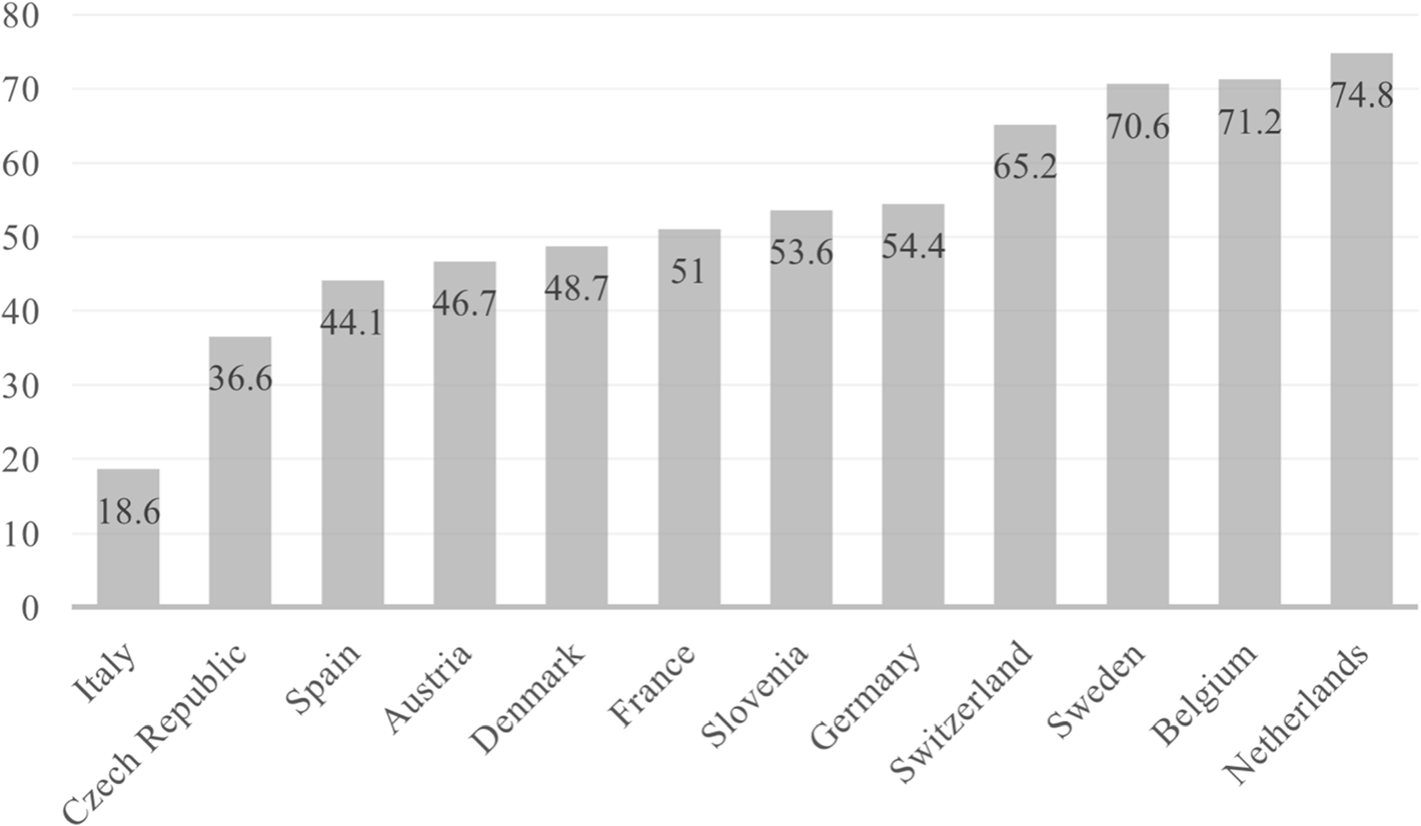
Beds in residential long-term care facilities (nursing homes) per 1000 population aged 65 and older, 2017. Data refer to 2011 for Denmark and 2012 for Belgium. Source: OECD. Stat Long-Term Care Resources and Utilization

A similar clustering of countries is found if one considers the number of beds available in nursing homes (called residential care facilities in OECD data (2020) by country (Fig. 2). On the one extreme, there is Italy with only 18 available beds for a thousand individuals older than 65; on the other, Sweden offers 70 beds, going up to 75 in the Netherlands.

Together with different levels of spending in LTC, and the availability of residential care facilities, also different eligibility criteria for nursing home admission are in place. All over Europe, reforms from the 90s aimed at containing the costs of institutional care increased the means-testing for nursing home admission with the goal to concentrate it to the cases of serious need (Pavolini & Ranci, 2008). Despite this common goal, a considerable polarization persists. Northern countries (Sweden, Denmark) maintain a de-familialising system (Albertini & Pavolini, 2015) where the public funding of long-term institutional care—but also other forms of support, such as cash benefits—is generous, even if means-tested on the level of economic resources. In Southern Europe, publicly funded residential care facilities are scarce, and care responsibilities remain mainly unloaded on the family (Geerts & van den Bosch, 2012) with the extreme case of the Italian LTC system, characterized by inertia and the unloading of family responsibilities on low-cost immigrant workers (Bettio, Simonazzi, & Villa, 2006). Spain slightly differs, as it adopts a cost-sharing mechanism, where the state contributes to residential care cost for around 80%, and the rest is on the shoulders of the individual, according to financial resources (Colombo et al., 2011); the availability of beds remains nonetheless limited (as showed in Fig. 2). Similarly, Eastern European countries (Slovenia and the Czech Republic) have a system where the care recipients are expected to cover full costs; exemption is available for very exceptional financial circumstances. Continental European countries lie somewhere in the middle, with country-specific cost-sharing arrangements between state and care recipient: in France, for example, care recipients cover residential care costs via housing allowance; in Belgium, health costs of nursing home residents are covered by the health insurance, while board and lodging costs are covered by the state via income means-testing; in the Netherlands, because of the attempt to disincentivize, the use of nursing home and incentivize home-based services, care recipients are expected to pay more than half of the costs (Alders, Costa-Font, de Klerk, & Frank, 2015).

To wit, the country of residence influences older individuals’ chances of living in a nursing home by different levels of public spending in long-term institutional care and availability of beds (Stolz, Mayerl, Rásky, & Freidl, 2019); but these chances might not be equal across population strata. Availability of beds in care facilities, together with means-tested measures, might make it easier for low-resource individuals to access care homes, creating social inequality in the probability of living in a nursing home, as we will delineate in the next paragraph.

### Disparities in living arrangements for the elderly

Despite the preferred option for frail individuals is aging in their own home (Eurobarometer, 2007; Filipovič Hrast, Sendi, Hlebec, & Kerbler, 2019), not everyone manages to do it. The availability of beds in nursing homes, as well as measures aimed at increasing the means-testing, could open avenues for inequalities in the composition of nursing home residents. For example, a large availability of beds in state-subsidized nursing homes, regulated by means-testing measures, may lead to a concentration of older people from a low socio-economic background in this kind of living arrangement.

Together with differences among institutional settings, also individual resources may play a crucial role in determining the probability of entering into a nursing home. On the one hand, relatives or friends may not be able to provide informal care, leaving older individuals to rely on formal support only (Bonsang, 2009). On the other hand, individuals of advanced age, with pre-existing health conditions (e.g., dementia, Parkinson and Alzheimer’s disease, see Agüero-Torres, Von Strauss, Viitanen, Winblad, & Fratiglioni, 2001), can be hardly handled at home (Luppa et al., 2009; Miller & Weissert, 2000), becoming the principal inhabitants of nursing homes. However, the conditions of loneliness in late life, as well as serious health impairment, are not equally distributed across the population. Low-SES individuals have worse health (Huisman, Kunst, & Mackenbach, 2003), and at the same time, less resources to finance their long-term care expenses at home (Bonnet, Juin, & Laferrère, 2019). Moreover, they are more likely to feel lonely (Vozikaki, Papadaki, Linardakis, & Philalithis, 2018) and to remain without a partner at younger ages (especially women, see (Mackenbach, Kunst, Cavelaars, Groenhof, & Geurts, 1997). For all these reasons (worst health conditions, less economic resources, and being more likely without a partner), they are likely to be eligible for means-tested measures supporting the LTC they need.

There is only a handful of studies investigating social inequality in the probability of living in a nursing home. Moving to a nursing home is more frequent for those in the lowest income quartile (Angelini & Laferrère, 2012; Laferrère et al., 2012), but cross-country differences in the gradient are not purposely investigated. Medical and gerontological research found no striking evidence for educational level differences (Luppa et al., 2009; Miller & Weissert, 2000), but these studies are often country-specific surveys limited in size and geographic reach.

## Data, variables, and method

In this study, we use the last three available waves (waves 5, 6, and 7; from 2013, 2015, and 2017, respectively) of the Survey of Health, Ageing, and Retirement in Europe (SHARE) (Börsch-Supan et al., 2013), which is a cross-national biannual longitudinal study collecting information on individuals older than 50 years old across 27 European countries and Israel. We restrict our analyses to individuals older than 65 years old and to those European countries which have a sufficient number of individuals living in a nursing home. After applying these restrictions, we are left with the following countries: Austria, Belgium, Czech Republic, Denmark, France, Germany, Italy, The Netherlands, Slovenia, Spain, Sweden, and Switzerland. For the Netherlands, we consider the most recent data available, in wave 5 (2013).

We pool data from waves 5, 6, and 7 and consider whether the respondents were living in a nursing home in any of the 3 years considered.

Our dependent variable is the probability of living in a nursing home instead than in a private household. In SHARE, nursing home are defined in the following way: “A nursing home provides all of the following services for its residents: dispensing of medication, available 24-h personal assistance and supervision (not necessarily a nurse), and room and meals” (Laferrère et al., 2012, p. 254). Therefore, the category “nursing home,” includes several forms of long-term care and it overlaps with the definition of residential care for older people provided by the OECD (2020).

We use two measures of socio-economic status as independent variables. The first one is couple-level household income. It is calculated as the sum of all income of the respondent and the spouse (if any) at the time of the first available survey, after any taxes and contributions. It includes earnings from employment, retirement pensions, unemployment or disability benefits and other forms of social assistance, and income from rent. Total household income is treated as a dichotomous variable, dividing individuals who have a household income below the country median, from individuals who have a household income above the country median. As a robustness check, we have replicated the analysis with household income equivalized at the couple level, following the approach of Albertini and Pavolini (2015). Results confirm the findings presented here, and we discuss them in the results section.

The second measure is the level of education of the respondent. Education is measured in SHARE with the International Standard Classification of Education (ISCED-1997) in 7 categories. The lowest category refers to individuals with less than primary education and the highest category to tertiary education or more. To make the assessment of the educational gradient in the risk of being in a nursing home comparable across countries, we use a strategy similar to Reardon (2011). We consider education as a latent characteristic in a population having a country-specific cumulative distribution (rank), which we can only observe with a measurement, ISCED-97 in this case. For each ISCED category, we compute the average percentile of the country-specific educational distribution, and we assign it to each individual. By doing so, we create the rank (the percentile) each observation is in the country-specific educational distribution. In this article, we consider as low educated individuals at the bottom 20th percentile (20p) of the country-specific educational distribution, and as high educated individuals those at the top 80th percentile (80p).

Missing values differ between our two independent variables. In order to maximize the number of observations, we use two (slightly different) analytical samples, one for each of the two independent variables. Sample 1 (S1) for income has *N*=37,528, while sample 2 (S2) for educational level has *N*=39,958.

Given the small absolute number of individuals living in a nursing home, to investigate socio-economic differences in the probability of residing in a nursing home, we estimate rare event logit models (Coveney, 2008). When the event of interest is rare, the conventional logit model tends to underestimate the probability of the event and produce a biased estimation of the coefficient of the explanatory variables (Firth, 1993). Rare event logit models are estimated with a penalized likelihood function that reduces the small-sample bias, separately for each country.

We also estimate our models controlling for gender, health status, and age. The decision on whether to control for age in our model is critical and must be discussed carefully. Age, as an indicator of a cohort of birth, is a possible confounder of the relationship between education and the probability of living in a nursing home. On the one hand, older people tend to be less educated and, on the other, older individuals have more need of support for personal care and are thus more likely to live in a nursing home. At the same time, however, education affects life expectancy so that low-educated individuals tend to have lower life expectancy than those who are highly educated. If low-educated individuals who reach older ages are positively selected on other typically unobserved characteristics, such as genetic predisposition to have good health, our results probably underestimate of the effect of low education on residential arrangement. In addition to being a possible confounder, age and health status are also possible colliders in the relationship between socio-economic status and the probability of living in a nursing home (Greenland, 2003; Schooling & Au Yeung, 2017). Other unobserved factors (such as for instance the place of residence) can affect life expectancy (and thus age) and health, and also the availability of access to institutional care services. For this reason, when we control for age and health status in our models, we are likely to introduce a collider bias in the estimates of the effect of income and education. In the absence of a straightforward solution, we consider the results of both specifications, controlling and not controlling for age and health status.

## Results

### Descriptive results

In Table 1, we present the distribution of our dependent and key independent variables. Table 1 reports two panels with descriptive statistics one for each sample (S1 and S2). Column 1 shows the percentage of those who have ever lived in a nursing home between 2013 and 2017 for both samples. In column 2, we display the percentage of individuals having a lower secondary education or less (ISCED 0/2 - column 2) for each country. For income, in column 2, the percentage of those with household income below the median is not reported, being 50% by definition in each country. In column 3, we report the share of nursing home residents having an income below the country median and having a lower secondary education or less. Overall, the share of individuals who have ever been nursing home residents is very low, although there are notable differences across countries (see column 1). In Western Europe, Belgium has the largest share of over 65 residing in a nursing home (about 6%), while Italy has the smallest (about 1%). The share of individuals in nursing homes estimated from SHARE matches the OCED official figures relatively well. The correlation between the percentages in Table 1 for 2017 and the number of beds ×1000 in nursing homes over the population aged 65 years old or more based on OECD 2017 data is far from perfect but reasonably high (*r*=0.65). Finally, column 3 in S1 and the comparison between columns 3 and 2 in S2 of Table 1 suggest that subjects who have an income below the country median or who are low educated are over-represented in nursing homes in all the countries considered, with respect to the relative size of their group in each country.

**Table 1:** Descriptive statistics by country for population aged 65 or more

**Sample 1 (S1): income**	(1)		(2)		(3)		(4)
	In a nursing home		% ≤ median income^a^		% ≤ median income in a nursing home		Total
	%	N			%	N	
*All countries*	3.19	1198			76.63	918	37,528
Austria	3.05	94			80.85	76	3079
Germany	2.55	77			75.32	58	3018
Sweden	3.28	107			83.18	89	3266
Netherlands	3.01	57			89.47	51	1892
Spain	2.62	124			75.00	93	4727
Italy	1.18	46			58.70	27	3887
France	3.58	109			80.73	88	3042
Denmark	4.30	96			88.54	85	2233
Switzerland	3.39	75			73.33	55	1909
Belgium	6.51	236			64.41	152	3625
Czech Republic	3.33	135			83.70	113	4052
Slovenia	1.50	42			73.81	31	2798
**Sample 2 (S2): education**	In a nursing home		% ≤ lower secondary education		% ≤ lower secondary education in a nursing home		Total
	%	N	%	N	%	N	
*All countries*	3.02	1208	47.37	18,928	61.92	748	39,958
Austria	2.96	94	26.97	858	42.55	40	3181
Germany	2.39	78	15.59	509	32.05	25	3264
Sweden	3.01	110	44.13	1612	60.91	67	3653
Netherlands	2.76	59	54.88	1175	79.66	47	2141
Spain	2.56	124	86.06	4161	91.94	114	4835
Italy	1.14	46	76.93	3095	78.26	36	4023
France	3.42	110	53.06	1706	75.45	83	3215
Denmark	3.94	97	23.73	584	54.64	53	2461
Switzerland	3.60	76	24.99	528	44.74	34	2113
Belgium	6.06	237	46.07	1801	61.60	146	3909
Czech Republic	3.26	135	41.44	1716	57.04	77	4141
Slovenia	1.39	42	39.15	1183	61.90	26	3022

*Source*: SHARE wave 5 (2013), 6 (2015), and 7 (2017). Own elaboration

The Netherlands is from wave 5 only

^a^By construction this variable splits the distribution into two

### Main results: the risk of living in a nursing home

**Fig. 3:**
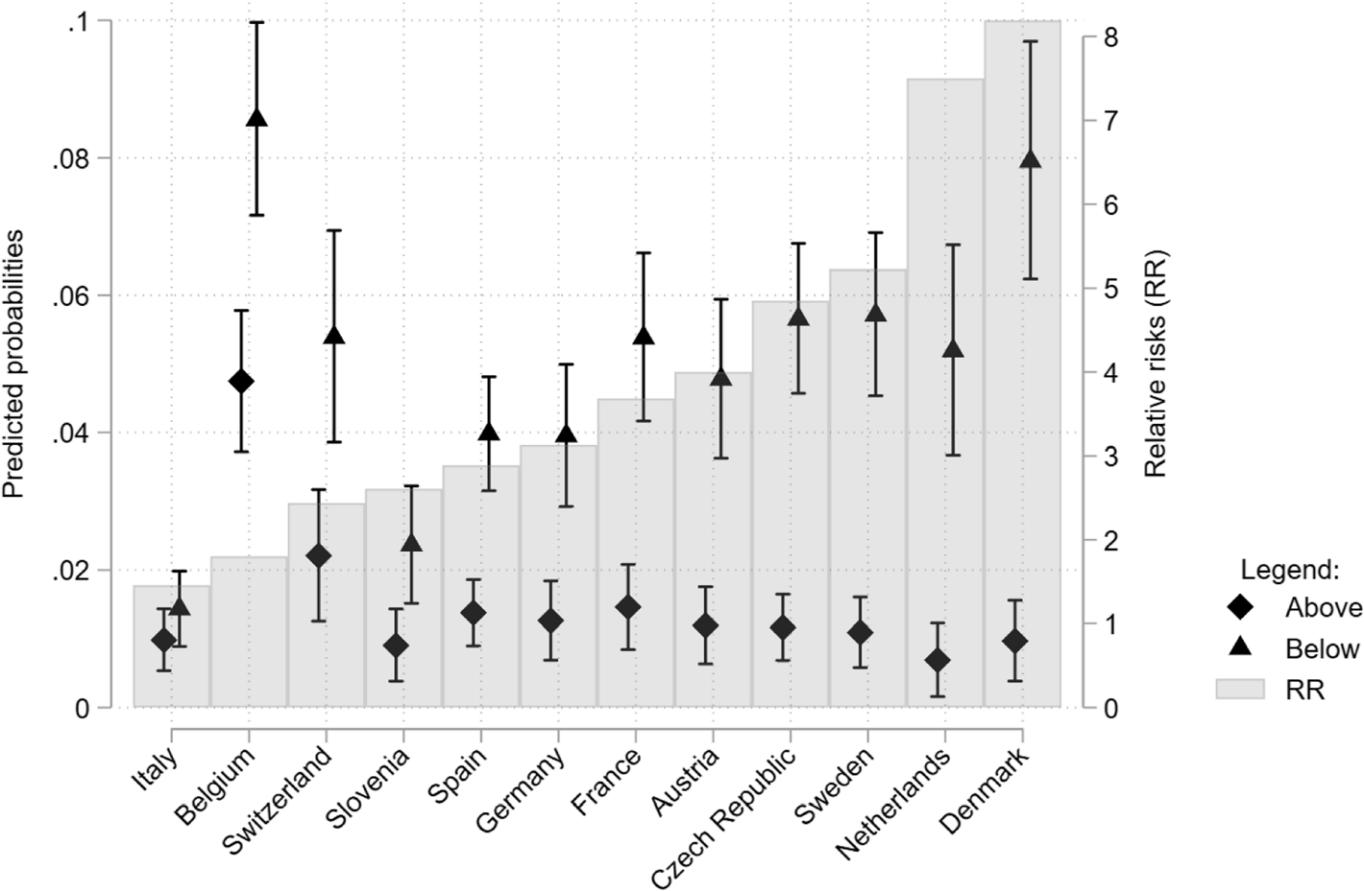
Predicted probabilities (left axis) and 95% confidence intervals for individuals below the median income (triangles) and above median income (diamonds) and associate relative risks (RR; bars; right axis) of being in a nursing home by country, controlling for respondents’ sex

Figure 3 reports the predicted probabilities (left-axis) and the relative risks (RR – right axis) of being a nursing home resident for those whose household income is below (triangles) the national median versus those above (diamonds), based on rare logit models estimated separately for each country, controlling only for the gender of the respondent. In all countries, we find that those with low household income have a higher probability of being in a nursing home compared to those who have an income above the national median. The confidence intervals of our point estimates are very large, given the small sample size in many countries, and a close comparison of effect sizes is not possible. In some countries, however, the relative risks are large and the probability of living in a nursing home for the low-income individuals is four to seven times (Denmark and the Netherlands) higher compared to high-income individuals. In Italy and Belgium, the relative risks are just above one, but while in Italy, the probabilities of living in a nursing home are small for both socio-economic groups, in Belgium, they are high for both of them.

**Fig. 4:**
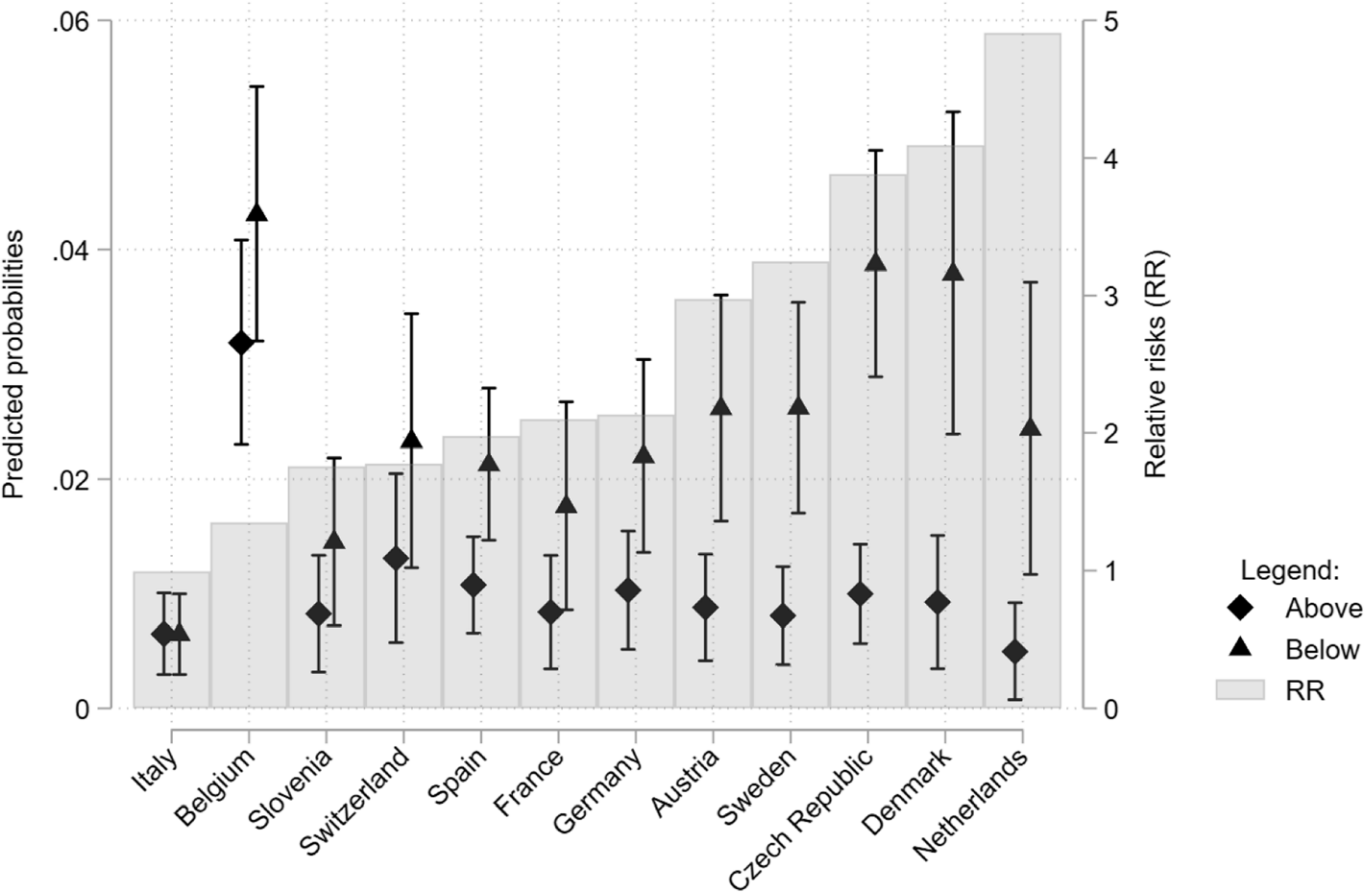
Predicted probabilities (left axis) and 95% confidence intervals for individuals below the median income (triangles) and above median income (diamonds) and associate relative risks (RR; bars; right axis) of being in a nursing home by country, controlling for respondent’s sex, age, and health status

In Fig. 4, we show the predicted probabilities and relative risks by income groups and country, after controlling for age and health status. The relative risks get reduced, suggesting that health status, in particular, mediates part of the observed association between household income and the probability of living in a nursing home. In Nordic countries (Sweden, Denmark), the Netherlands, and the Czech Republic, the relative risks remain large in these countries that the probability of living in a nursing home is three to four times larger compared to high-income individuals. In the other countries, with the already noted exception of Italy and Belgium, the confidence intervals of the predicted probabilities overlap and the difference in the predicted probabilities for the low and high educated are not statistically significant. We interpret the absence of statistical significance as evidence of considerable uncertainty around the point estimates but not as an indication of no socio-economic differences (Bernardi, Chakhaia, & Leopold, 2017). In particular, the results for countries such as Germany, Spain, and France still point that there are socio-economic inequalities in the probability of living in a nursing home.

**Fig. 7:**
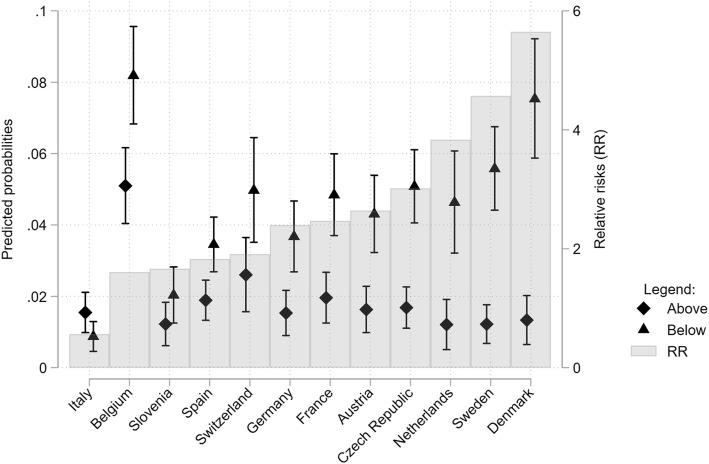
Predicted probabilities (left axis) and 95% confidence intervals for individuals below the median income (triangles) and above-median equalized income (diamonds) and associate relative risks (RR; bars; right axis) of being in a nursing home by country controlling for respondent’s sex

**Fig. 8:**
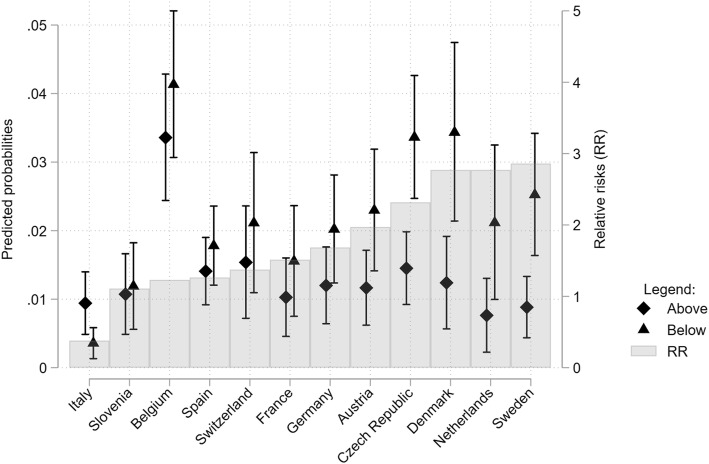
Predicted probabilities (left axis) and 95% confidence intervals for individuals below the median income (triangles) and above-median equalized income (diamonds) and associate relative risks (RR; bars; right axis) of being in a nursing home by country, controlling for age and health status

In Appendix Figure 5 and 6, we show the relative risks by education and in Figure 7 and 8 by equivalized income at the couple level. We also report the odds ratios for both education and household income in Appendix Table 2. Overall, the results for education and the equivalized income confirm the general finding that the probability of living in a nursing home is higher for low socio-economic groups. Despite a few exceptions, they also confirm the pattern of cross-country differences found for income with a more pronounced inequality in Nordic countries and The Netherlands, and no inequality on the opposite in Italy. When controlling for age and health status, the reduction in the relative risks income is more marked, particularly in the case of education, but the overall pattern of results is confirmed. At the same time, this latter finding suggests the confounding effect of age is more pronounced in the case of education and income.

## Conclusions

In this study, we investigated the socio-economic composition of nursing home residents in order to assess the stratified risk of residing in one of the most critical trouble spots of the COVID-19 pandemic. Our analyses show that low-income and low-educated individuals are more likely to live in nursing homes. This result is found across the 12 countries included in our study, with the partial exception of Italy. In Italy, in fact, the LTC system is characterized by a limited diffusion of institutional care services and the responsibility of the care of elderly population is unloaded to their families with the support of cash programs (Albertini & Pavolini, 2015; Jessoula et al., 2018). In most of the countries, we find ratios higher than two, suggesting that differences in the probability of living in a care home by income are sizeable. There is, however, considerable uncertainty in our estimates due to the small sample size and firm conclusions on how the effect of education and income varies across countries cannot be drawn. Still, there is some indication that the largest educational and income differences are found in the Nordic countries (Denmark and Sweden) and the Netherlands, and the smallest ones in Italy, with other countries laying in between. This pattern seems to indicate that where there is more availability of beds, means-testing, and more people receive formal care, those with disadvantaged backgrounds are more likely to be in nursing homes. The result partially contrasts another study on the topic (Albertini & Pavolini, 2015) which finds more stratification in Italy than in Denmark when considering *formal care* use tout-court (nursing home placement and home-based services together). In Italy, home-based services and cash programs supporting families make the most of the LTC services provided by the welfare state. When considering the whole LTC services mix, therefore, it is likely that a socio-economic gradient is also found in Italy.

Once we control for age and health status, the relative risks get smaller, but the general pattern is still confirmed, indicating that disadvantaged individuals have a higher probability of living in a nursing home. We have also noted that age and health status are a collider of the relationship between the indicators of socio-economic status and the probability of living in a nursing home, and that including age and health status as a control variable is likely to bias the estimates of the effect of education and income (Greenland, 2003). This is a complex methodological issue that we could not adequately address in this manuscript.

With all due cautiousness due to the small number of cases and large confidence intervals for many countries, our central finding holds important implications for those interested in social inequality and concerned with public policy in the COVID-19 pandemics, more generally. Given the extremely high death Toll in care homes, evidence on socio-economic differences in the probability of being in nursing homes could provide evidence, although indirect, of social inequality in the risk of mortality linked to COVID-19.

Moreover, the finding that low socio-economic groups are disproportionally present in nursing homes and thus more exposed to the pandemic also raises issues of social justice. Guaranteeing adequate protection against infection in the nursing homes is a paramount issue not only of public health in reducing the scope of the pandemic, but also of social policy toward inequality reduction.

To conclude, the present study is not without limitations. Firstly, SHARE data do not allow to distinguish between public and private nursing homes. Information on the private LTC market is overall scarce (see Colombo et al., 2011), as the phenomenon is gaining importance only in recent years in European countries such as Germany, France, and Czech Republic (Eurofound, 2017). Surely, a higher share of private nursing homes in a country could alter the pattern of inequality in the composition of the residents, even though the direction is not straightforward. While more advantaged individuals could be more able to buy private service, older individuals from lower social strata could benefit from public subsidies in private institutions (such as compulsory health insurances) (*ibidem)*. Secondly, the sample size, given the low proportion of the older population living in a nursing home, even in a survey on individuals 50+ like SHARE. It would, then, be desirable to replicate our findings using administrative data or ad hoc large surveys on nursing home access and their residents for those countries where such data are available and/or freely accessible. Second, despite our interest in COVID-related mortality inequality, disaggregated data on mortality in nursing homes because of COVID were not available for European countries. Therefore, we could not provide direct evidence that mortality in a nursing home has contributed to the socio-economic gradient in mortality during the COVID 19 crisis. Despite this shortcoming, we believe that higher incidence of residents in nursing homes who are low-educated and low-income that we have documented adds another worrying element to the already dramatic finding of the spike of deaths in a nursing home for COVID 19: those living where COVID 19 has caused what has been called a “silent massacre” disproportionally come from the most disadvantaged socio-economic groups.

## Figures and Tables

**Fig. 5:**
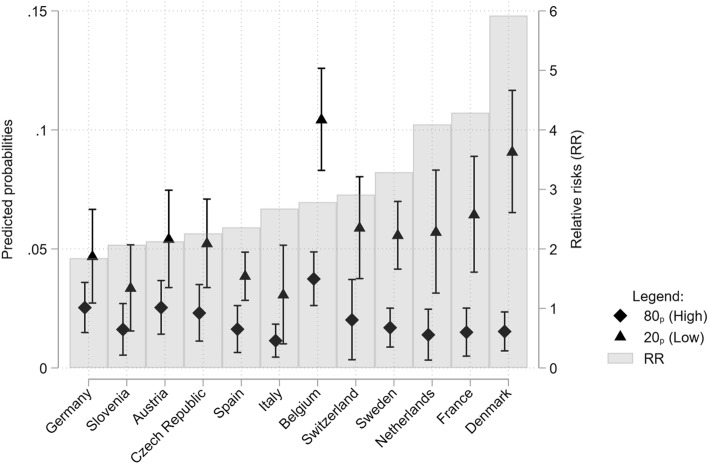
Predicted probabilities (left axis) and 95% confidence intervals for low educated (triangles) and high educated (diamonds) and associate relative risks (bars; right axis) of being in a nursing home by country, controlling for respondent’s sex

**Fig. 6:**
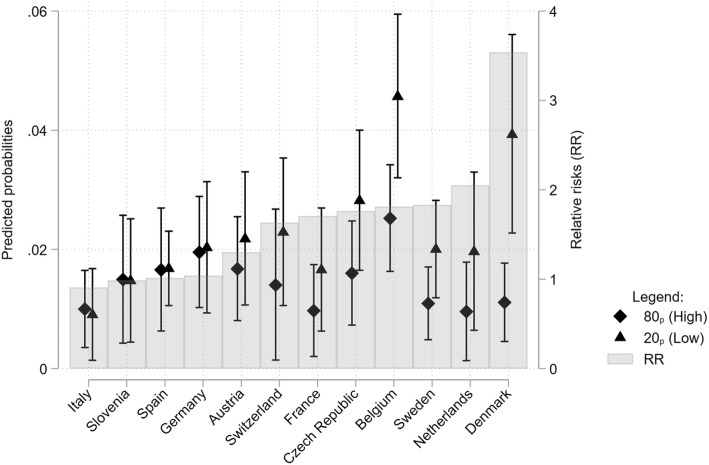
Predicted probabilities (left axis) and 95% confidence intervals for low educated (triangles) and high educated (diamonds) and associate relative risks (bars; right axis) of being in a nursing home by country, controlling for respondent’s sex, age, and health status

## Data Availability

This paper uses data from SHARE Waves 5, 6, and 7 (DOIs: 10.6103/SHARE.w5.710, 10.6103/SHARE.w6.710, 10.6103/SHARE.w7.710), see Börsch-Supan et al. (2013) for methodological details. Data access is publicly granted upon completion of the application form: http://www.share-project.org/data-access/user-registration.html.

## Appendix

**Table 2:** Odds ratios of being in a nursing home by couple household income and education

Country	Income				Education			
	Non-adjusted		Age and health adjusted		Non-adjusted		Age and health adjusted	
	OR	SE	OR	SE	OR	SE	OR	SE
Austria	3.89***	1.03	2.89***	0.78	2.16***	0.65	1.30	0.40
Germany	2.95***	0.78	2.01***	0.55	1.87**	0.59	1.04	0.34
Sweden	5.06***	1.31	3.12***	0.85	3.36***	0.94	1.84**	0.55
Netherlands	7.67***	3.23	4.95***	2.10	4.18***	1.93	2.06	0.98
Spain	2.93***	0.61	2.01***	0.43	2.40**	0.81	1.01	0.35
Italy	1.33	0.40	0.92	0.28	2.70**	1.26	0.91	0.45
France	3.99***	0.98	2.31***	0.58	4.40***	1.75	1.71	0.70
Denmark	7.63***	2.43	3.73***	1.24	6.16***	1.91	3.59***	1.16
Switzerland	2.58***	0.68	1.87**	0.51	2.97**	1.41	1.64	0.81
Belgium	1.76***	0.25	1.31*	0.19	2.88***	0.55	1.83***	0.36
Czech Rep.	5.08***	1.20	4.13***	0.99	2.30**	0.74	1.77	0.58
Slovenia	2.65***	0.93	1.85*	0.67	2.09*	0.93	0.99	0.47

Models include respondent’s sex as control variable. Significance level: **p* value < 0.10; ***p* value < 0.05; ****p* value < 0.01

## Footnotes

1 ^a^From now onward, we will refer to nursing homes to include both type of accommodations

2 https://www.politico.eu/article/the-silent-coronavirus-covid19-massacre-in-italy-milan-lombardy-nursing-care-homes-elderly/

## References

[CR1] Agüero-Torres H, Von Strauss E, Viitanen M, Winblad B, Fratiglioni L (2001). Institutionalization in the elderly: the role of chronic diseases and dementia. Cross-sectional and longitudinal data from a population-based study. Journal of Clinical Epidemiology.

[CR2] Albertini M, Pavolini E (2015). Unequal inequalities: the stratification of the use of formal care among older Europeans. Journals of Gerontology Series B: Psychological Sciences and Social Sciences.

[CR3] Alders P, Costa-Font J, de Klerk M, Frank R (2015). What is the impact of policy differences on nursing home utilization? The cases of Germany and the Netherlands. Health Policy.

[CR4] Angelini V, Laferrère A (2012). Residential mobility of the European elderly. CESifo Economic Studies.

[CR5] Bernardi F, Chakhaia L, Leopold L (2017). ‘Sing Me a Song with Social Significance’: the (Mis) use of statistical significance testing in European Sociological Research. European Sociological Review.

[CR6] Bettio F, Simonazzi A, Villa P (2006). Change in care regimes and female migration: the “care drain” in the Mediterranean. Journal of European Social Policy.

[CR7] Bonnet C, Juin S, Laferrère A (2019). Private financing of long term care: income, savings and reverse mortgages. Economie et Statistique / Economics and Statistics.

[CR8] Bonsang E (2009). Does informal care from children to their elderly parents substitute for formal care in Europe?. Journal of Health Economics.

[CR9] Börsch-Supan A, Brandt M, Hunkler C, Kneip T, Korbmacher J, Malter F (2013). Data Resource Profile: The Survey of Health, Ageing and Retirement in Europe (SHARE). International Journal of Epidemiology.

[CR10] Colombo, F., Llena-Nozal, A., Mercier, J., & Tjadens, F. (2011). Help wanted? Providing and paying for long-term care. In *OECD Health Policy Studies*. OECD Publishing. 10.1787/9789264097759-en.

[CR11] Coveney, J. (2008). FIRTHLOGIT: Stata module to calculate bias reduction in logistic regression. In *Statistical Software Components S456948*. Boston College Department of Economics.

[CR12] Danis, K., Fonteneau, L., Georges, S., Daniau, C., Bernard-Stoecklin, S., Domegan, L., … Vandael, E. (2020). High impact of COVID-19 in long-term care facilities, suggestion for monitoring in the EU/EEA, May 2020. *Eurosurveillance*, *25*(22), 2000956. 10.2807/1560-7917.ES.2020.25.22.2000956 PMC7336111 32524949

[CR13] ECDC (2020). Increase in fatal cases of COVID-19 among long-term care facility residents in the EU/EEA and the UK. 19 November 2020.

[CR14] Eurobarometer (2007). *Health and long-term care in the European Union (Wave 67.3)*. Special Eurobarometer 283.

[CR15] Eurofound (2017). Care homes for older Europeans: public, for-profit and non-profit providers, Publications Office of the European Union, Luxembourg.

[CR16] Filipovič Hrast M, Sendi R, Hlebec V, Kerbler B (2019). Moving house and housing preferences in older age in Slovenia. Housing, Theory and Society.

[CR17] Firth, D. (1993). Biometrika trust bias reduction of maximum likelihood estimates. *Biometrica*, *80*(1), 27–38. 10.1093/biomet/80.1.27.

[CR18] Frijters, S., & van Uffelen, X. (2020). *Helft van de coronasterfte in Europa vindt plaats in verpleeghuizen*. De Volkskrant Retrieved from https://www.volkskrant.nl/nieuws-achtergrond/helft-van-de-coronasterfte-in-europa-vindt-plaats-in-verpleeghuizen~b7922f60/.

[CR19] Geerts, J., & van den Bosch, K. (2012). Transitions in formal and informal care utilisation amongst older Europeans: the impact of national contexts. *European Journal of Ageing*, *9*(1), 27–37. 10.1007/s10433-011-0199-z. 10.1007/s10433-011-0199-z PMC5547315 28804405

[CR20] Gori, C., Fernandez, J.-L. W., & R. (2015). *Long-term care reforms in OECD countries*. Policy Press. 10.1332/policypress/9781447305057.001.0001.

[CR21] Greenland, S. (2003). Quantifying biases in causal models: classical confounding vs collider-stratification bias. *Epidemiology*, *14*(3), 300–306. 10.1097/01.EDE.0000042804.12056.6C. 12859030

[CR22] Haberkern, K., Schmid, T., Neuberger, F., & Grignon, M. (2011). The role of the elderly as providers and recipients of care. In *The Future of Families to 2030*, (pp. 189–257).

[CR23] Huisman, M., Kunst, A. E., & Mackenbach, J. P. (2003). Socioeconomic inequalities in morbidity among the elderly; a European overview. *Social Science and Medicine*, *57*(5), 861–873. 10.1016/S0277-9536(02)00454-9. 10.1016/s0277-9536(02)00454-9 12850111

[CR24] Laferrère, A., Van Den Heede, A., & Van Den Bosch, K. (2012). Entry into institutional care: predictors and alternatives. In A. Börsch-Supan, M. Brandt, H. Litwin, & G. Weber (Eds.), *Active Ageing and Solidarity between Generations in Europe*, (pp. 253–264). De Gruyter.

[CR25] Lipszyc, B., Sail, E., & Xavier, A. (2012). Long-term care: need, use and expenditure in the EU-27. *European Economy Economic Papers*, *469*. 11.

[CR26] Luppa M, Luck T, Weyerer S, König HH, Brähler E, Riedel-Heller SG (2009). Prediction of institutionalization in the elderly. A systematic review. Age and Ageing.

[CR27] Mackenbach JP, Kunst A, Cavelaars A, Groenhof F, Geurts J (1997). Socioeconomic inequalities in morbidity and mortality in western Europe. Lancet.

[CR28] Jessoula, M., Pavolini E., Raitano, M., Natili, M. (2018). *ESPN thematic report on challenges in long-term care*.

[CR29] Miller EA, Weissert WG (2000). Predicting elderly people’s risk for nursing home placement, hospitalization, functional impairment, and mortality: a synthesis. Medical Care Research and Review.

[CR30] OECD (2020). OECD Health Statistics 2020. Definitions, sources and methods: beds in residential long-term care facilities.

[CR31] Orange, R. (2020). *Anger in Sweden as elderly pay price for coronavirus strategy*. The Guardian Retrieved from https://www.theguardian.com/world/2020/apr/19/anger-in-sweden-as-elderly-pay-price-for-coronavirus-strategy.

[CR32] Pavolini, E., & Ranci, C. (2008). Restructuring the welfare state: reforms in long-term care in Western European countries. *Journal of European Social Policy*, *18*(3), 246–259. 10.1177/0958928708091058.

[CR33] Reardon SF (2011). The widening academic achievement gap between the rich and the poor: new evidence and possible explanations. Whither opportunity.

[CR34] Schooling, C. M., & Au Yeung, S. L. (2017). “Selection bias by death” and other ways collider bias may cause the obesity paradox. *Epidemiology*, *28*(2), 16–17. 10.1097/EDE.0000000000000591 27922527

[CR35] Stolz, E., Mayerl, H., Rásky, É., & Freidl, W. (2019). Individual and country-level determinants of nursing home admission in the last year of life in Europe. *PLoS ONE*, *14*(3), 1–10. 10.1371/journal.pone.0213787 PMC6417724 30870521

[CR36] Strang P, Fürst P, Schultz T (2020). Excess deaths from COVID-19 correlate with age and socio-economic status. A database study in the Stockholm region. Upsala Journal of Medical Sciences.

[CR37] Vozikaki M, Papadaki A, Linardakis M, Philalithis A (2018). Loneliness among older European adults: results from the survey of health, aging and retirement in Europe. Journal of Public Health: From Theory to Practice.

